# Persistent Infection in Harbor Seals 12–13 Years after Phocine Distemper Virus Epizootics in 1988 and 2002, North Sea

**DOI:** 10.3201/eid3112.250329

**Published:** 2025-12

**Authors:** Marco W.G. van de Bildt, Jolianne M. Rijks, Trine Hammer Jensen, Sophie M.J.M. Brasseur, Marja J.L. Kik, Albert D.M.E. Osterhaus, Andrea Gröne, Thijs Kuiken, Jooske IJzer

**Affiliations:** Erasmus University Medical Centre, Rotterdam, the Netherlands (M.W.G. van de Bildt, T. Kuiken); Dutch Wildlife Health Centre, Utrecht, the Netherlands (J.M. Rijks, M.J.L. Kik, J. IJzer); Aalborg University, Aalborg, Denmark (T. Hammer Jensen); Wageningen Marine Research, Den Helder, the Netherlands (S.M.J.M. Brasseur); Utrecht University, Utrecht (M.J.L. Kik, A. Gröne, J. IJzer); University of Veterinary Medicine, Hannover, Germany (A.D.M.E. Osterhaus)

**Keywords:** phocine distemper virus, Morbillivirus, viruses, harbor seal, Phoca vitulina, old seal encephalitis, the Netherlands

## Abstract

Phocine distemper virus caused epizootics of fatal pneumonia in North Sea harbor seals in 1988 and 2002. Two seals that stranded years later were infected with defective phocine distemper virus variants that caused severe encephalomyelitis. Old seal encephalitis resembled subacute sclerosing panencephalitis in humans and old dog encephalitis in canines.

Phocine distemper virus (PDV) epizootics among harbor seals (*Phoca vitulina*) in Europe caused the deaths of »23,000 seals in 1988 and »30,000 in 2002 (*1*). Infected animals had marked lymphoid depletion and necrosis, broncho-interstitial pneumonia, and, in some cases, nonsuppurative encephalitis (*2*). For other members of the genus *Morbillivirus*, family *Paramyxoviridae*, including measles virus of humans and canine distemper virus of dogs, rare occasions of persistent central nervous system (CNS) infections have been reported that caused neurologic signs 3–20 years after the primary infection (*3*,*4*), characterized by nonsuppurative encephalitis, demyelination, and intranuclear inclusion bodies, and result in subacute sclerosing panencephalitis (SSPE) in humans and old dog encephalitis (ODE) in dogs. We report comparable persistent PDV in harbor seals, for which we propose the name old seal encephalitis.

## The Study

We found 2 harbor seals from the North Sea stranded alive along the Netherlands coast in 2001 and 2014. The stranding of the first seal (case 1) was 13 years after the 1988 PDV epizootic and of the second seal (case 2) was 12 years after the 2002 PDV epizootic. We admitted the first seal for rehabilitation but had to euthanize it 3 weeks later because of progressive paresis of its hind limbs and weight loss. After postmortem examination, we estimated its age as 19 years, based on cementum layers in one of its upper canine teeth (*5*). We estimated the second seal as 15 years of age based on cementum layers; the animal was stranded during an influenza A(H10N7) outbreak. We euthanized it on the day of stranding because of emaciation and apathy.

We performed postmortem examinations on seal carcasses and collected samples of major organs for pathologic and virologic analyses. We performed pathologic analysis on tissues fixed in 10% neutral-buffered formalin and embedded in paraffin blocks. We cut sections 4 microns thick and stained them with hematoxylin and eosin or with luxol fast blue for histopathologic examination. We stained sequential tissue sections for virus antigen detection by an immunohistochemical technique, either using a monoclonal canine distemper virus antibody known to cross-react with PDV (VMRD, https://www.vmrd.com) for morbillivirus antigen detection or a hemagglutinin-specific mouse monoclonal antibody HB65 (ATTC H16-L10-4R5) for influenza virus antigen detection.

We performed virologic analysis for morbillivirus as described previously (*6*). In brief, we used tissue samples that had been stored frozen to test for morbillivirus by reverse-transcription PCR (RT-PCR) using universal morbillivirus primers, based on conserved sequences in the phosphoprotein, hemagglutinin (HA), or matrix (M) genes (*7*). We sequenced selected HA gene fragments for phylogenetic comparison. To isolate morbillivirus, we inoculated 10% tissue homogenate samples of seal brain (case 1 and case 2), liver, spleen, kidney, and urinary bladder (case 2) onto Vero.DogSLAMtag cells (*8*). We made 3 consecutive passages and checked for cytopathic changes daily. We tested cells for the presence of morbillivirus by immunofluorescence (IFA) (*9*) and tested cells and supernatants by RT-PCR. We also tested tissue samples for influenza A virus by RT-PCR using specific primers (*10*).

**Figure 1:**
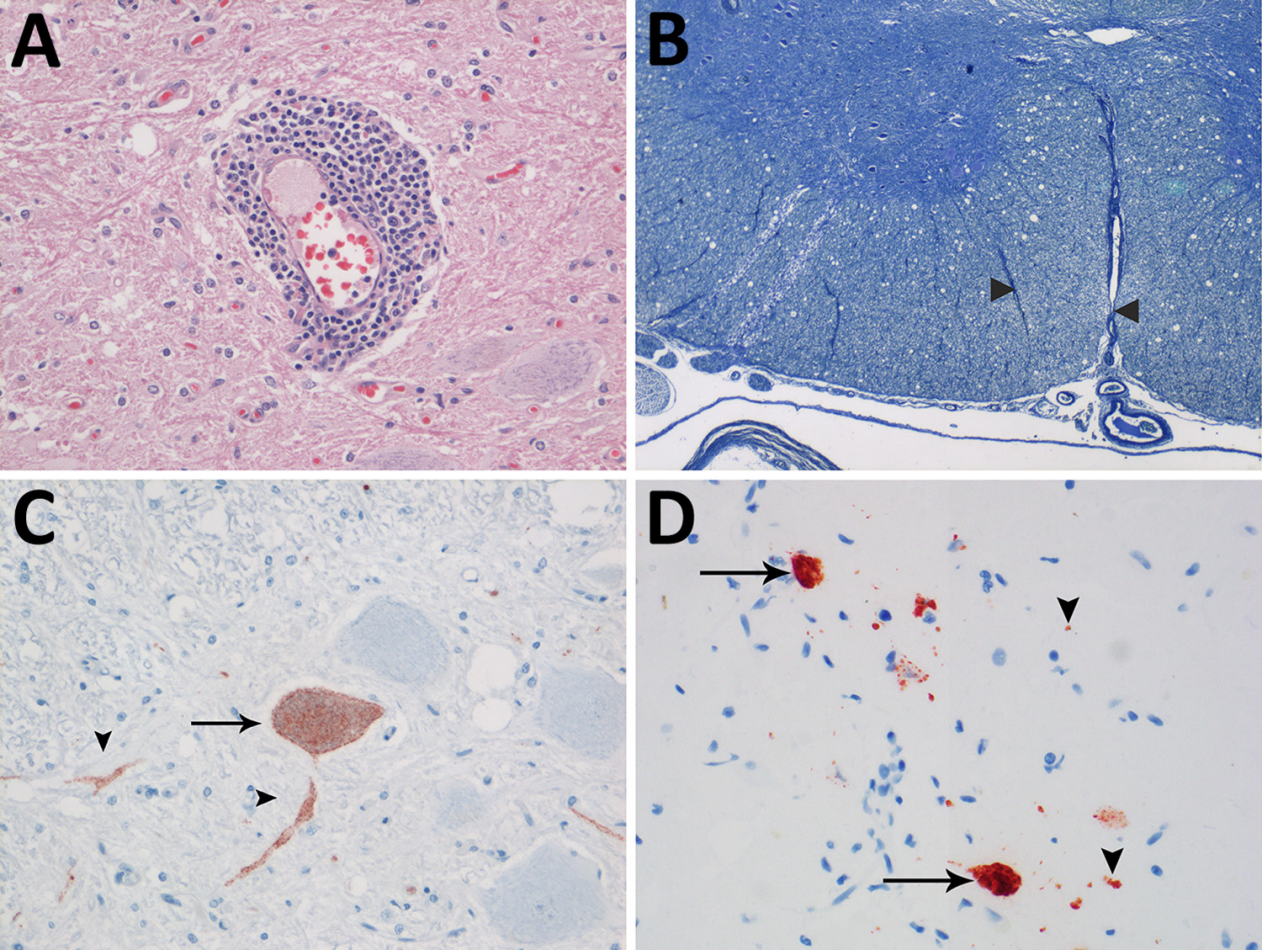
Evidence of persistent infection in 2 harbor seals stranded in the North Sea region years after phocine distemper virus epizootics. A) Marked perivascular accumulation of lymphocytes and plasma cells stained with hematoxylin and eosin from a seal infected in the 1988 epizootic outbreak (case 1). Original magnification ×200. B) Vacuolation and demyelination (between arrowheads) in the cervical spinal cord in case 1, stained with Kluver luxol fast blue. Original magnification ×20. C) Phocine distemper virus antigen expression in neuronal cell body (arrows) and axons or dendrites (arrowheads) of the spinal cord in case 1 by immunohistochemistry using monoclonal antibody against canine distemper virus. Original magnification ×400. D) Phocine distemper virus antigen expression in neuronal cell bodies (arrows) and axons or dendrites (arrowheads) of the spinal cord of a seal infected in the 2002 epizootic outbreak (case 2) by immunohistochemistry using monoclonal antibody against canine distemper virus. Original magnification ×400.

**Figure 2:**
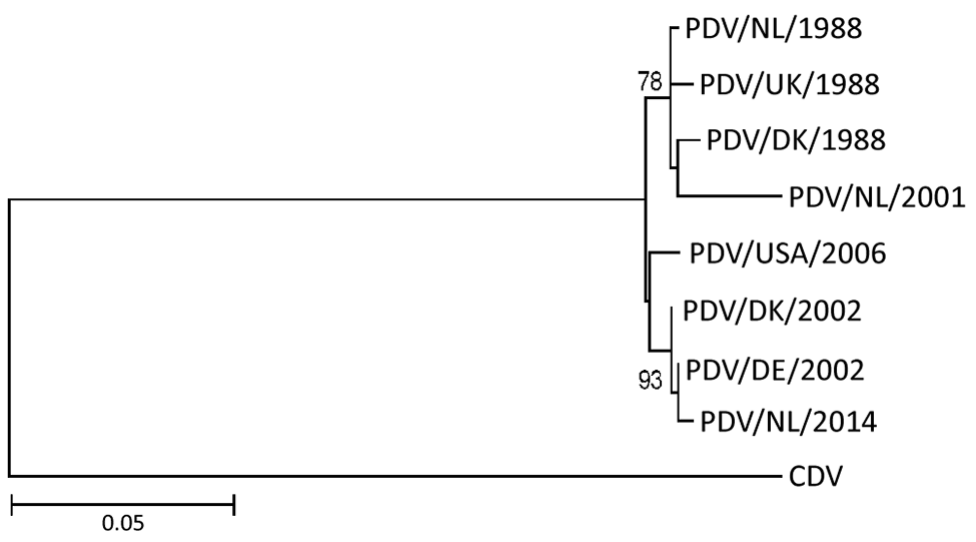
Phylogenetic maximum-likelihood tree based on the deduced amino acid sequence of the hemagglutinin gene (1824 nt) in study of persistent infection in 2 harbor seals stranded in the North Sea region years after phocine distemper virus epizootics. Tree was constructed using the Jones-Taylor-Thornton substitution model with invariant sites and gamma distributed rate variation with 1,000 bootstrap replicates in MEGA 6.0 (https://www.megasoftware.net). Bootstrap values >70% are shown at nodes. GenBank accession numbers for each isolate were as follows: PDV/NL/1988, NC 028249.1; PDV/UK/1988, D10371.1; PDV/DK/1988, Z36979.1; PDV/USA/2006, HQ007902.1; PDV/DK/2002, FJ648456.1; PDV/DE/2002, KU342692.1; CDV, NC 001921.1. Isolates from this study were deposited under accession nos. KY681679.1 (PDV/NL/2001) and KU342688.1 (PDV/NL/2014). Scale bar indicates nucleotide substitutions per site. CDV, canine distemper virus; PDV, phocine distemper virus.

We observed no gross lesions in brain, spinal cord, or sciatic nerves of either seal during postmortem examination. Both seals had multifocal nonsuppurative encephalomyelitis with perivascular lymphoplasmacytic cuffing (Figure 1, panel A) and rare neuronal necrosis, characterized by shrunken cells, hypereosinophilic cytoplasm, and loss of nuclei. We found lesions in the cerebrum of both seals and the cerebellum and spinal cord in case 1. In case 1, we observed demyelination in cerebellum and in ventral (Figure 1, panel B) and lateral funiculi of the spinal cord, whereas the dorsal funiculi were relatively spared. In case 2, demyelination was absent. We did not observe inclusion bodies in either case. Immunohistochemistry for morbillivirus showed staining of neurons at the sites of CNS lesions (Figure 1, panels C, D). We detected no other major lesions in case 1. In case 2, we detected avian influenza A(H10N7) virus in the lungs by RT-PCR. We did not detect influenza A antigen in the cerebrum, lung, or pulmonary lymph node in case 2. We detected the presence of PDV in the brains, but not lungs, of both animals by RT-PCR for morbillivirus and sequencing of the amplicon. We designated the strains PDV/NL/2001 (case 1) and PDV/NL/2014 (case 2). Phylogenetic analysis of the HA gene revealed that PDV/NL/2001 was most closely related to the virus from 1988, whereas PDV/NL/2014 clustered together with PDV isolated during the outbreak of 2002 (Figure 2). The M and HA genes of both PDV strains had a region with numerous uracil-to-cytosine substitutions; we identified 19 substitutions in the cytoplasmic domain and the anchor sequence of the HA gene of PDV/NL/2001 and 5 substitutions in those of PDV/NL/2014. We also observed in PDV/NL/2014 a putative truncation of the M gene. We were unable to isolate morbillivirus from tissue samples and detected no morbillivirus with IFA or RT-PCR.

## Conclusions

In this study, we demonstrated marked multifocal nonsuppurative encephalomyelitis associated with PDV infection in 2 harbor seals that stranded several years after PDV epizootics had occurred. The seal in case 1 would have been 6 years of age during the PDV epizootic in the Northeast Atlantic harbor seal population in 1988; it was stranded in 2001 (13 years later) with an infection of PDV that clustered with those from the 1988 epizootic. The seal in case 2 would have been 3 years of age during the second PDV epizootic in 2002; it was stranded in 2014 (12 years later) with an infection of PDV that clustered with those from the 2002 epizootic. In the years after the PDV epizootics in 1988 and 2002, PDV antibody prevalence progressively declined in the Northeast Atlantic harbor seal population, indicating that PDV was no longer circulating in the population (*1*,*11*). Taking those results together, the simplest explanation is that the case 1 seal was infected during the 1988 and the case 2 seal during the 2002 PDV epizootic.

The viruses involved had multiple mutations in the HA and M genes, which resemble the biased hypermutation observed in measles virus sequences from SSPE cases (*12*). Together with the putative truncation of the M gene observed in PDV/NL/14, those mutations might result in aberrant viral protein expression. Those genetic changes, together with the failure to isolate PDV from tissue samples of cases 1 and 2, are consistent with the presence of defective and noninfectious viruses in the brains of those seals as described for persistent measles virus and canine distemper virus in the brain (*3*,*13*).

We conclude that PDV can cause persistent infection in the brains of harbor seals, with a pathogenesis similar to that of SSPE in humans from persistent measles virus infection and ODE in domestic dogs from persistent canine distemper virus infection. The characteristics of the 2 cases in harbor seals fit with those of ODE in domestic dogs and SSPE in humans in the timing (several years after presumed infection), organ distribution (limited to the CNS), level of inflammation (marked perivascular aggregation of lymphoid cells), presence of viral inclusions (variable frequency, absent in some cases), and virus properties (defective, noninfectious virus) (*3*,*4*). The presence of typical lesions in the spinal cord in case 1 is different from the distribution of lesions of ODE, which are typically restricted to the forebrain (*4*). We found no previous evidence for an ODE-like syndrome in PDV infections of pinnipeds (*14*). In line with the nomenclature in dogs, we propose the name old seal encephalitis. 

The effect of PDV on the North Sea harbor seal population was thought to be limited to the periods of the 1988 and 2002 epizootics. However, the persistent infections of PDV in harbor seals causing severe neurologic disease implied that its effect extends into the interepizootic period. Our findings suggest a potential for delayed-onset encephalitis (i.e., SSPE) as a result of the current measles epidemic in the United States and Europe (*15*).
